# Ketamine Augmentation of Electroconvulsive Therapy: A Scoping Review of Dose-Dependent Effects in Major Depressive Disorder

**DOI:** 10.7759/cureus.40087

**Published:** 2023-06-07

**Authors:** Tarika Nagi, Amit Jagtiani, Saurabh Somvanshi, Satesh A Seegobin, Jasbir Singh, Anil K Bachu, Meenal Pathak

**Affiliations:** 1 Psychiatry, Columbia University College of Physicians and Surgeons, Harlem Hospital Center, New York, USA; 2 Psychiatry, Burrell Behavioral Health, Springfield, USA; 3 Psychiatry and Behavioral Sciences, Northwell Health - Zucker Hillside Hospital, New York, USA; 4 Psychiatry, Ross University School of Medicine, Bridgetown, BRB; 5 Psychiatry, University of California, Los Angeles - Kern Medical Center, Bakersfield, USA; 6 Psychiatry and Behavioral Sciences, Baptist Health-UAMS (University of Arkansas for Medical Sciences), Little Rock, USA; 7 Psychiatry and Behavioral Sciences, Allegheny Health Network, Pittsburgh, USA; 8 Psychiatry, Penn State Health Milton S. Hershey Medical Center, Hershey, USA

**Keywords:** i.v. ketamine, ketamine with ect, refractory depression, treatment-resistant depression, depression ketamine, iv ketamine, ketamine anesthesia, depression, electroconvulsive therapy (ect), ect anesthesia

## Abstract

Intravenous ketamine infusions in subanesthetic doses have been shown to rapidly alleviate depressive symptoms. However, the efficacy of ketamine as an anesthetic during electroconvulsive therapy (ECT) for major depression has not yet been answered by a large randomized control trial (RCT). This scoping review aims to examine the available literature to determine whether the dose of ketamine used during ECT influences the response to treatment. A literature search was conducted on PubMed to identify all published RCTs within the last 10 years which compared ketamine anesthesia during ECT for major depression with another anesthetic. Studies using low (<0.8 mg/kg) versus high (≥0.8 mg/kg) doses of ketamine during ECT were evaluated for the differences in outcomes using depression rating scales. Studies that examined ketamine as a standalone treatment for depression or focused primarily on the anesthetic benefits of ketamine were excluded from our review. Fifteen studies were utilized for this literature review. Overall, the studies showed inconsistent results in terms of the speed and magnitude of response to ketamine-assisted ECT in patients with major depression. Limitations of the available literature are discussed, including the lack of head-to-head comparisons, differences in methodology, inclusion/exclusion criteria, and primary and secondary endpoints.

## Introduction and background

Major depressive disorder (MDD) is a highly prevalent and complex illness with various etiologies that affect populations of all demographics. In acute care inpatient settings, one of the most common consultations in consultation-liaison psychiatry is depression [1]. Depression and suicidal ideation are also highly prevalent in the pediatric population where bullying and adverse childhood events are key risk factors [2]. Interestingly, electroconvulsive therapy (ECT) is a preferred treatment in those who experience nihilistic delusions with resistant cases of MDD as they are at increased risk of self-harm and suicidal behavior [3]. Despite the availability of more than 25 FDA-approved medications for MDD, treatment-resistant depression (TRD) remains prevalent, with approximately 30% of patients failing to achieve remission after multiple trials of first-line antidepressants [4]. Although ECT is an effective treatment for severe depression, its usage is limited due to side effects (transient confusion, short-term memory loss, and cognitive impairment), the need for hospitalization, and stigma [5].

Recent years have seen a growing interest in the use of ketamine, a non-competitive N-methyl-D-aspartate receptor antagonist, as a novel and rapidly acting treatment for MDD. Studies have also been investigating the use of various adjunctive treatments with ECT such as lithium in TRD with interesting results about the ketamine group improving the Montgomery-Asberg depression rating scale (MADRS) score more than the midazolam group 24 hours after treatment [6,7]. Intravenous ketamine infusions in subanesthetic doses have been shown to have a rapid antidepressant effect, with some studies reporting symptom improvement as early as one to two hours post-infusion [8]. This has led to investigations into the potential use of ketamine in conjunction with ECT for MDD. While ECT is an effective treatment for depression, its use can be limited by the cognitive side effects and the time required for repeated treatments [9]. Using ketamine anesthesia during ECT, it may be possible to enhance the antidepressant effects of ECT while also reducing cognitive side effects and treatment time. However, the optimal dose of ketamine for use during ECT remains unclear. The purpose of this scoping review is to examine the existing literature on the use of ketamine during ECT for MDD and to evaluate whether different doses of ketamine influence treatment outcomes.

## Review

Methodology

**Figure 1:**
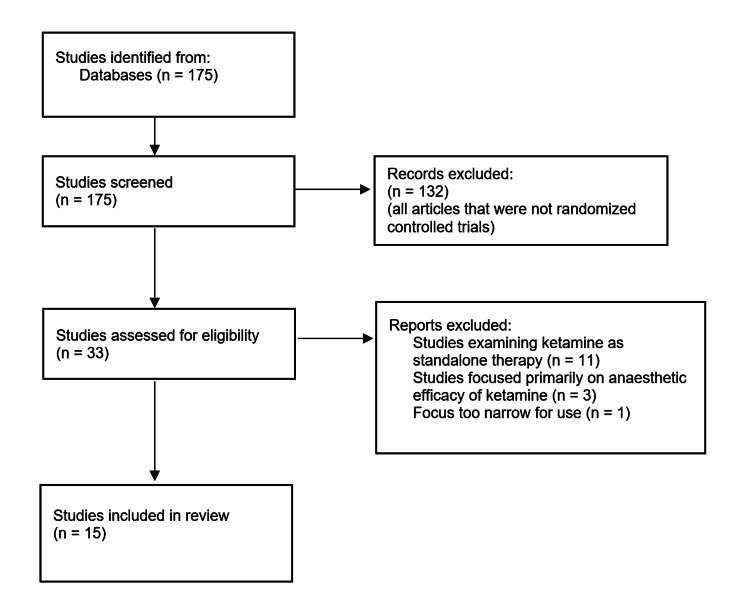
Criteria for selecting the studies included in this review

A literature search was conducted on PubMed using the search terms "ketamine," "ECT," and "depression" to identify all published studies within the last 10 years which compared ketamine anesthesia during ECT for major depression with another anesthetic. Studies using low (<0.8 mg/kg) versus high (≥0.8 mg/kg) doses of ketamine during ECT were evaluated for differences in outcomes using depression rating scales. All studies that were included in the review were randomized controlled trials (RCTs) that measured depression improvement using a rating scale throughout ECT administration with ketamine anesthesia. All studies focusing on ketamine as a standalone treatment for depression or comparing ketamine treatment individually to ECT were excluded from the study. A total of 15 articles were selected for review that met our criteria as described in Figure 1.

Three depression rating scales were used to assess the severity of depression in the included studies: the Beck Depression Inventory (BDI), the Hamilton depression rating scale (HDRS), and the MADRS. The BDI is a self-administered questionnaire that consists of 21 items assessing the symptoms of depression, with each item scored on a scale of 0-3 [10]. The HDRS is a clinician-administered questionnaire that assesses 17 items related to depression severity, with each item scored on a scale of 0-2 or 0-4 [11]. The MADRS is a clinician-administered questionnaire that assesses the severity of depression based on 10 items, with each item scored on a scale of 0-60 [12]. The use of different scales was noted as a limitation in the study as they may not be directly comparable due to differences in the number and types of items assessed.

Our literature search identified a total of 15 studies that examined the effects of ketamine anesthesia during ECT for major depression. However, comparing the studies directly proved to be challenging due to the heterogeneity of the studies, including differences in depression rating scales used, dosages and types of anesthetics given, number of treatments of ECT administered, and measurement of magnitudes of responses. As a result, a head-to-head comparison of the studies was not possible. Instead, we reviewed each study individually to evaluate whether there were differences in outcomes between studies using low (<0.8 mg/kg) versus high (≥0.8 mg/kg) doses of ketamine during ECT for major depression. Despite these efforts, limitations remained, including the varying inclusion and exclusion criteria, primary and secondary endpoints, and the different depression rating scales used in each study.

Low-dose ketamine anesthesia in ECT for TRD

The effectiveness of low-dose ketamine as an adjunct to ECT in the treatment of severe depression has been investigated in several studies. In a randomized study conducted by Järventausta et al. [13], the researchers aimed to assess whether the administration of a low dose of ketamine prior to ECT sessions could enhance its antidepressant effects. The study included participants diagnosed with MDD or bipolar disorder who were undergoing ECT for a major depressive episode. The participants were randomly assigned to receive either thiopental alone or thiopental with ketamine (0.5 mg/kg) for anesthesia before each ECT session. The severity of depression was measured using the HDRS at baseline and after the first and sixth ECT sessions. Both groups demonstrated significant antidepressant effects from ECT, indicating the efficacy of the treatment in reducing depressive symptoms. However, no significant difference was observed between the two groups in HDRS scores following the first ECT session. These findings suggest that administering ketamine at a dose of 0.5 mg/kg before ECT does not enhance the antidepressant effects of treatment. Similarly, in a parallel-group randomized study conducted by Zhang et al. [14], the efficacy of ketamine (0.5 mg/kg) as an adjunctive treatment during ECT for patients with severe depression was investigated. The study included 79 participants diagnosed with unipolar or bipolar depression who were randomly assigned to receive either ketamine or saline. However, the results of this study did not demonstrate any benefits of ketamine over saline in routine ECT treatment.

Another study conducted by Woolsey et al. [15] investigated the effects of S-ketamine (0.4 mg/kg) against normal saline as an adjuvant to propofol anesthesia used in 32 patients undergoing ECT for TRD. No significant differences were found between the two groups when assessing response to ECT treatments; however, the study found that the S-ketamine group experienced increased levels of disorientation and restlessness post-treatment. Despite these findings, the study concluded that subanesthetic doses of S-ketamine in combination with propofol do not enhance the treatment efficacy of ECT in patients with TRD. These findings align with a study conducted by Alizadeh et al. [16], which included 77 inpatients with MDD (n = 41) and bipolar disorder (n = 36). The participants were randomly assigned to receive ECT with propofol anesthesia (1.0 mg/kg) or a combination of ketamine (0.5 mg/kg) and propofol (0.5 mg/kg). Depressive symptoms were assessed using the HDRS and MADRS rating scales before and after ECT treatments and during the one to four weeks following the last treatment. Cognitive performance was evaluated using a battery of tests at baseline, after the sixth ECT treatment, and during the one to four weeks following the final ECT treatment. The study found no significant differences in depressive symptoms, cognitive performance, or adverse effects between the treatment groups at any time. It was noted in this study that the electrical dose required to induce seizures in the ketamine plus propofol group was lower compared to the propofol-only group. They also found that seizure durations were increased in the ketamine-propofol group as compared to the propofol group. The study concluded that the addition of ketamine to propofol anesthesia during ECT for MDD and bipolar disorder did not show superiority over propofol alone. Corroborating these findings, Zou et al. found that low-dose ketamine anesthesia did not improve psychiatric outcomes as an adjunct to propofol-based anesthesia in a study involving 45 patients and did not reduce the number of ECT sessions required to reduce MADRS scores by 25%-50% [17].

Loo et al. [18] also investigated the differences observed when administering ketamine and propofol together as anesthesia compared to propofol alone in a double-blind RCT involving 42 patients with MDD. The severity of depression was assessed using the HDRS before ECT was administered and at a two-week follow-up appointment. Though both groups exhibited a reduction in depression severity, there was no significant difference observed between the groups. This study could not demonstrate a significant effect of ketamine on depression recovery within the two-week follow-up period; however, the ketamine group showed a shorter recovery time with respect to improvement in cognitive performance post-treatment compared to the control group, suggesting that ketamine may have a positive impact on cognitive function in patients undergoing ECT. Carspecken et al. [19] similarly found that low-dose ketamine (0.3 mg/kg) when combined with propofol anesthesia decreased the rate of cognitive impairment in patients receiving ECT for TRD. Kuşçu et al. [20] explored whether ketamine (0.5 mg/kg) reduced cognitive impairment and enhanced the efficacy of depression treatment during the course of ECT in a study involving 51 depressed patients undergoing right unilateral ECT. They were randomly assigned to receive either ketamine (0.5 mg/kg) or normal saline (placebo) in addition to thiopentone during anesthesia for ECT. There were no significant differences in neuropsychological outcomes between the ketamine and placebo groups. Though no overall difference in efficacy was observed at the end of the ECT course, the ketamine-ECT group showed a slightly greater improvement in depressive symptoms during the first week of treatment indicating that ketamine might accelerate recovery from depression compared to alternative anesthetics.

High-dose ketamine anesthesia in ECT for TRD

A randomized, double-blinded clinical trial conducted by Ray-Griffith et al. [21] investigated the effects of ketamine on brain-derived-neurotrophic-factor (BDNF) and treatment outcomes in patients undergoing ECT for TRD. The patients with TRD received either methohexital (1-2 mg/kg) or ketamine (1-2 mg/kg) anesthesia during ECT. Depression severity was measured using self-reported and clinician-assessed questionnaires before treatments began and after the final session was completed. No significant differences were observed between either group with respect to seizure length or intensity. No significant difference was observed in depression scores of both groups. Of note, 15% of participants in the methohexital group failed to achieve adequate seizures and required a switch to ketamine anesthesia, while 26% required conversion to bilateral ECT stimulus. It was found that all ketamine patients achieved adequate seizures, and only 4% required bilateral stimulus. Additionally, ketamine was found to increase serum levels of BDNF and did not increase the rates of post-ECT agitation.

Another study by Yen et al. [22] included three groups of patients who received different anesthesia: Group 1 received thiopental (4 mg/kg), Group 2 received ketamine (1 mg/kg), and Group 3 received a combination of ketamine (1 mg/kg) and thiopental (4 mg/kg). Succinylcholine (1 mg/kg) was used in all patients for muscle relaxation. The study measured HDRS and Hamilton anxiety rating scale (HAM-A) scores, seizure duration, and hemodynamic variables before and after anesthesia administration and after the ECT procedure. There was a reduction in HDRS scores compared to baseline values in all groups, but no significant difference was observed between the groups. HAM-A scores were higher in Group 2 and Group 3. The study concluded that anesthesia induced with the thiopental, ketamine, and thiopental-ketamine combination did not produce a difference in ECT outcomes for patients with TRD. The administration of ketamine at a dose of 1 mg/kg immediately before ECT did not enhance the antidepressant effect of the treatment and increased anxiety levels in patients.

A study that compared the effects of ketamine and methohexital as primary anesthetic agents during ECT for depression reported similar findings with respect to the impact on the HDRS score of patients. The study by Fernie et al. [23] included 21 subjects with unipolar or bipolar depression. Both ketamine and methohexital groups showed significant improvement in depressive symptoms measured by the HDRS over time, with no statistical difference between the two groups. However, subjects receiving ketamine reported more fatigue compared to the methohexital group. The results were inconclusive, suggesting no advantage of ketamine anesthesia over methohexital in alleviating depressive symptoms during ECT. Rybakowski et al. also found that ketamine at a dose of 1 mg/kg was associated with increased subject drop-out rate, reorientation time, and negative side effects (nausea, dysphoria, and dizziness) with no observed reduction in post-anesthesia recovery time [24]. Yoosefi et al. compared the effects of ketamine (up to 2 mg/kg) with intravenous propofol (up to 2.5 mg/kg) and found no significant differences in the treatment number, memory impairment, and improvement in depression severity ratings either during or one month after the ECT course [25].

One study found that ketamine might enhance the antidepressant effect of ECT treatments in patients with TRD. Salehi et al. [26] investigated the use of ketamine as an anesthetic in ECT, looking into its antidepressant effects on the treatment and potential impact on the post-treatment cognition of each patient involved. The study included 45 patients aged 21-75 years who were diagnosed with MDD. These patients were then divided into three groups: Group 1 received thiopental alone; Group 2 received ketamine along with thiopental for the second and third ECT sessions; and Group 3 received ketamine along with thiopental for the second, fourth, sixth, eighth, and 10th sessions. The HDRS scale was used to measure depression severity, and cognitive assessments were conducted before and after ECT treatments to evaluate visual-spatial abilities, working memory, verbal-auditory memory, and executive functions. At baseline, the HDRS score for the patients involved in the study was 32 points (SD = 6) with no significant difference observed between the three groups. Following the final ECT session, a significant reduction in HDRS scores was observed in Group 3 compared to Group 1. Of note, cognitive assessments after ECT treatments demonstrated a greater decline in verbal memory in the patients who received ketamine anesthesia. This study concluded that while ketamine may enhance the antidepressant efficacy of ECT, it could potentially have a negative impact on verbal memory post-treatment.

Wang et al. [27] presented conflicting results where they found that ketamine improved cognitive function significantly compared to thiopental. This randomized, double-blind clinical trial included 29 patients with MDD who were scheduled for ECT and randomly assigned to receive either ketamine or thiopental anesthesia. The mini-mental state examination was used to assess memory, and the HDRS was used to assess depression. Each patient underwent a total of six ECT sessions. Both groups showed improvement in HDRS score though a significant difference in depression improvement was observed only before the second ECT session in the ketamine group. Despite a significant decline in the mini-mental state examination scores in both groups after the first ECT session, cognitive function improved significantly in the ketamine group post-treatment. Seizure duration was found to be significantly longer with ketamine. The stimulus intensity used for each ECT session gradually and linearly increased, with a greater increase observed in the thiopental group. The researchers concluded that ketamine administration during ECT is well-tolerated, and patients may experience earlier improvement in depressive symptoms, longer seizure duration, and better cognitive performance compared to thiopental. Zhong et al. [28] also compared ketamine and thiopental anesthesia with mixed findings. A total of 160 patients with TRD were randomly assigned to two groups: One group received ketamine at a dose of 0.8 mg/kg, and the other group received sodium thiopental at 1.5 mg/kg. The researchers evaluated seizure duration, recovery time, and anesthesia-related side effects one hour after the administration of anesthesia. Depression levels were assessed using the HDRS, and data regarding recovery time and complications were obtained during ECT sessions 2, 4, 6, and 8. The incidence of complications such as nausea, headaches, pain at the injection site, short-term delirium, and long-term delirium was found to be higher in the ketamine group (p > 0.05) as compared to those who received sodium thiopental alone. Despite the fact that there was no significant difference in depression scores at the end of the ECT course of treatments between these two groups, it was found that those who received ketamine experienced a more rapid rate of recovery from their depression compared to those who received sodium thiopental.

Rasmussen et al. [29] reported similar findings, suggesting that a combination of ketamine and propofol might result in earlier improvements in HDRS scores compared to those who receive propofol anesthesia alone. The study included 48 patients with HDRS scores greater than 20, who were randomly assigned to three groups: the propofol group (group P), the ketamine group (group K), and the propofol plus ketamine group (group PK). Propofol was administered at a dose of 1.5 mg/kg, ketamine at a dose of 0.8 mg/kg, and the combination of propofol and ketamine at the same doses. Both group K and group PK had earlier improvements in HDRS scores compared to group P. The decreases in HDRS scores were significantly greater in group K and group PK compared to group P. Group PK experienced fewer adverse effects than Group K. These findings suggest that a combination of ketamine and propofol might be the preferred choice of anesthetic for TRD patients undergoing ECT. Youssef et al. [30] also found that a combination of ketamine and propofol anesthesia resulted in earlier improvements in HDRS scores for patients with TRD in a study comparing the effects of anesthetic and subanesthetic concentrations of ketamine on mood, cognitive impairment, and seizure parameters. Ninety patients with TRD (36 males, 54 females; average age: 30.6 years) were randomly assigned to receive ketamine (0.8 mg/kg) (n = 30), subanesthetic ketamine (0.5 mg/kg) plus propofol (0.5 mg/kg) (n = 30), or propofol (0.8 mg/kg) (n = 30) as an anesthetic and underwent eight ECT sessions. The primary outcome measures included the HDRS, cognitive assessments, and seizure parameters. The group administered ketamine at anesthetic doses (0.8 mg/kg) exhibited earlier improvements in HDRS scores, longer seizure duration, a higher remission rate, and a lower degree of executive cognitive impairment compared to the ketamine plus propofol and propofol groups. The ketamine plus propofol group also showed earlier improvements in the HDRS scores and longer seizure duration compared to the propofol group. The researchers determined that anesthetic concentrations result in larger improvements in depression and cognitive protection. This conflicts with the findings by Patel et al. who found that ketamine anesthesia did not accelerate the recovery from depression or decrease cognitive side effects compared to methohexital when used during ECT in his study of 38 patients with TRD (Table 1) [31].

**Table 1:** Review of studies comparing the efficacy of ketamine anesthesia during ECT treatment of depression ND: No difference; K: Ketamine; P: Propofol; PK: Propofol + Ketamine; G1/G2/G3: Group 1/Group 2/Group 3; ECT: Electroconvulsive therapy.

			Speed of Response		Magnitude of Response		
Author	Comparison	Subject Numbers	Depression Scores and Response Rate at Mid Treatment	Number of ECTs to Remission	Response Rate	Remission Rate	Depression Scores at Completion
Abdallah et al., 2012 [11]	#Thiopental (3.5 mg/kg), #Thiopental (3.5 mg/kg) + ketamine (0.5 mg/kg)	N = 9, N = 9	ND		ND		ND
Anderson et al., 2017 [12]	Propofol/Thiopentone w/ #Ketamine (0.5 mg/kg), #Saline	N = 37, N = 33	ND	ND	ND	ND	ND
Järventausta et al., 2013 [13]	#Propofol + ketamine (0.4 mg/kg), #Propofol + saline	N = 16, N = 16	ND	ND	ND		
Zhang et al., 2018 [14]	#Propofol (1 mg/kg), #Propofol (1 mg/kg) + ketamine (0.5 mg/kg)	N = 34, N = 43	ND		ND	ND	ND
Alizadeh et al., 2015 [16]	#Propofol + ketamine (0.3 mg/kg), #Propofol + saline	N = 22, N = 20	ND				ND
Loo et al., 2012 [18]	#Thiopental + ketamine (0.5 mg/kg), #Thiopental + saline	N = 22, N = 24	ND (except at week 1)	ND	ND	ND	ND
Fernie et al., 2017 [23]	#Ketamine (up to 2 mg/kg), #Propofol (up to 2.5 mg/kg)	N = 20, N = 20	ND	ND			ND
Carspecken et al., 2018 [19]	#Ketamine (1-2 mg/kg), #Methohexital (1-2 mg/kg)	N = 23, N = 27					ND
Salehi et al., 2015 [26]	#Ketamine (0.8 mg/kg), #Thiopental (1.5 mg/kg)	N = 78, N = 75	No difference in depression scores after ECT 2,4,6				ND
Yoosefi et al., 2014 [25]	#Ketamine (1-2 mg/kg), #Thiopental (2-3 mg/kg)	N = 17, N = 14	Better depression scores after 1^st^ ECT in ketamine (didn’t measure after 3^rd^)				ND
Ray-Griffith et al., 2017 [21]	#Ketamine (1 mg/kg), #Methohexital (1 mg/kg)	N = 8, N = 8	No difference in depressive scores after ECT 1,2,3,4,5	ND	ND	ND	ND
Wang et al., 2012 [27]	#Propofol (1.5 mg/kg), #Ketamine (0.8 mg/kg), #Propofol (1.5 mg/kg) + ketamine (0.8 mg/kg)	N = 16, N = 16, N = 16	Better depression scores in K and PK groups				ND
Zhong et al., 2016 [28]	#Propofol (0.8 mg/kg) = P, #Ketamine (0.8 mg/kg) = K, #Propofol (0.5 mg/kg) + ketamine (0.5 mg/kg) = PK	N = 30, N = 30, N = 30	K > PK > P better depression scores from 2^nd^ to 8^th^ ECT and better response rate after ECT 3 and 4		ND	K > PK > P after 8^th^ ECT	K > PK > P
Kuşçu et al., 2015 [20]	#Thiopental (4 mg/kg), #Ketamine (1 mg/kg), #Thiopental (4 mg/kg) + ketamine (1 mg/kg)	N = 21, N = 19, N = 18	No difference in depression scores after 3^rd^ and 6^th^ ECT				ND
Rybakowski et al., 2016 [24]	#G1: Thiopentone (2-3 mg/kg), #G2: ketamine (1-1.5 mg/kg) [2,3], #G3: Ketamine (1-1.5 mg/kg) (during ECT 2,4,6,8)	N = 15, N = 15, N = 15	No difference in depression scores after 5^th^ ECT	ND			Lower in G3 compared to G1 and G2

Overall, the studies showed inconsistent results in terms of the speed and magnitude of response to ketamine-assisted ECT in patients with major depression. Low-dose ketamine studies consistently showed no significant advantage of ketamine anesthesia in improving the speed or magnitude of response in patients with depression. This is in contrast with the proven evidence of rapid antidepressant effects of low-dose ketamine infusions. High-dose ketamine studies showed inconsistent results in terms of speed and magnitude of response, with a few studies showing more rapid response with ketamine-assisted anesthesia.

Discussion

The role of ketamine as anesthesia in ECT for the treatment of MDD has been a topic of significant interest in recent years. In this study, we conducted a literature review to evaluate whether the dose of ketamine used during ECT determines the response of subjects to the treatment. Our review of the literature found that low-dose ketamine studies consistently showed no advantage of ketamine anesthesia in improving the speed or magnitude of response in patients with depression. This contrasts with the proven evidence of rapid antidepressant effects of low-dose ketamine infusions. One possible explanation for this discrepancy could be the adjuvant use of thiopental or propofol, which is well known to have anticonvulsant effects, potentially reducing the efficacy of ECT on depressive symptoms. On the other hand, high-dose ketamine studies showed inconsistent results in terms of the speed and magnitude of response, with a good number of studies showing a more rapid response with ketamine-assisted anesthesia. High-dose ketamine anesthesia in ECT might be more effective in achieving early remission of depression in high-risk situations like suicidality. One hypothesis for this could be that the higher doses of ketamine act on different pathways that are not activated by lower doses. Further studies are needed to evaluate this hypothesis and clarify the underlying mechanisms of action.

There were some limitations to our scoping review. Due to differences in methodology, inclusion/exclusion criteria, primary and secondary endpoints, and the use of different rating scales, a head-to-head comparison of studies was not possible. Nevertheless, our scoping review provides an overview of the current literature and highlights the need for future studies to address these limitations and further investigate the potential of ketamine-assisted anesthesia in ECT for the treatment of MDD.

Further research would require a robust design in which the effects of ketamine anesthesia in the ECT setting can appropriately be measured and studied. A large-scale RCT with adequate sample sizes should be utilized to detect the differences in outcomes between groups. It is also necessary for future studies to standardize ECT and ketamine administration in such a way that the type and frequency of ECT, dose of ketamine, and timing of ketamine administration are controlled for. The same depression rating scale should be used for all patients enrolled in the study, and follow-up assessments should take place at regular intervals after completion of the ECT course. A mechanistic study of ketamine anesthesia might also be useful to examine the effects of ketamine on neuroplasticity, the glutamate system, and inflammatory markers, which have been implicated in the pathophysiology of depression. By incorporating these methodological improvements, future studies can more accurately determine the effects of ketamine anesthesia during ECT for depression leading to improved treatment options for patients with TRD.

## Conclusions

In conclusion, while the use of ketamine as anesthesia during ECT for the treatment of MDD is still an area of active research, our review suggests that it is still unclear whether the dose of ketamine has a significant effect on the efficacy of ECT in the treatment of depression. The results from multiple studies evaluating the use of ketamine as an adjunct to ECT for TRD are mixed. Some studies found no significant difference in depression improvement when ketamine was used compared to other anesthetic agents or placebo, while others found a slight improvement in depressive symptoms in the first week of treatment or after a certain number of ECT sessions. However, most studies did not find any significant benefit of ketamine in enhancing the efficacy of ECT for TRD. Some studies even reported adverse effects of ketamine, such as increased anxiety scores and cognitive impairment. Therefore, overall, the results do not support the routine use of ketamine as an adjunct to ECT for TRD. Further research is needed to clarify the mechanisms of action of ketamine and to identify patient populations that may benefit the most from this treatment approach.
